# ARS: AI-Driven Recovery Controller for Quadruped Robot Using Single-Network Model

**DOI:** 10.3390/biomimetics9120749

**Published:** 2024-12-10

**Authors:** Han Sol Kang, Hyun Yong Lee, Ji Man Park, Seong Won Nam, Yeong Woo Son, Bum Su Yi, Jae Young Oh, Jun Ha Song, Soo Yeon Choi, Bo Geun Kim, Hyun Seok Kim, Hyouk Ryeol Choi

**Affiliations:** 1 Department of Mechanical Engineering, Sungkyunkwan University, 2066, Seobu-ro, Jangan-gu, Suwon 16419, Republic of Korea; kanghs0822@skku.edu (H.S.K.);; 2 AIDIN ROBOTICS Inc., Anyang 14055, Republic of Korea; 3 Department of Intelligent Robotics, Sungkyunkwan University, 2066, Seobu-ro, Jangan-gu, Suwon 16419, Republic of Korea; 4 Hyundai-Rotem Co., Uiwang 16082, Republic of Korea; hyunseok0427@hyundai-rotem.co.kr

**Keywords:** legged robot, reinforcement learning, fall recovery

## Abstract

Legged robots, especially quadruped robots, are widely used in various environments due to their advantage in overcoming rough terrains. However, falling is inevitable. Therefore, the ability to overcome a falling state is an essential ability for legged robots. In this paper, we propose a method to fully recover a quadruped robot from a fall using a single-neural network model. The neural network model is trained in two steps in simulations using reinforcement learning, and then directly applied to AiDIN-VIII, a quadruped robot with 12 degrees of freedom. Experimental results using the proposed method show that the robot can successfully recover from a fall within 5 s in various postures, even when the robot is completely turned over. In addition, we can see that the robot successfully recovers from a fall caused by a disturbance.

## 1. Introduction

Because legged robots are beneficial in that they can overcome various environments, including stairs and gaps that existing wheeled robots have difficulty navigating, research and development have been conducted around the world for the past several decades.

Successful locomotion of legged robots has been achieved over the last few decades by simplifying the robot into a Single-Rigid Body Model (SRBD) and utilizing Quadratic Programming (QP) or Model-Predictive Control (MPC) [1,2]. In addition, research on a Whole-Body Controller (WBC), which fully exploits robot’s dynamics without simplifying the robot system, has been actively conducted and has shown impressive results [3,4]. However, control using simplified models has limitations in complex environments, and developing a mathematically accurate model for the robot system is very challenging. On the other hand, substantial research has been conducted on controlling robots by integrating Artificial Intelligence (AI).

Tan et al. [5] conducted a study on quadruped robots’ locomotion using Reinforcement Learning (RL) with a robot called Minitaur from Ghost Robotics [6]. To address the sim-to-real problem in which neural networks trained in simulation do not perform well in real robots, they applied ideal DC motor dynamics modeling and a piece-wise torque–current relationship as an actuator model in the simulation. They also applied a method to randomize physical parameters, including latency. Hwangbo et al. [7] conducted research using ANYMal [8], a quadruped robot equipped with a Series Elastic Actuator (SEA). Because SEA is very difficult to model mathematically, applying an actuator model that models dynamics like [5] was challenging. They proposed a method of modeling an SEA using a neural network and applied it to a simulation, significantly reducing torque errors between the simulation and actual robots. Their approach demonstrated great potential for controlling legged robots using reinforcement learning.

Research on legged robots using reinforcement learning continues to expand, encompassing studies that utilize exteroceptive information [9,10], combine with model-based methods [11,12], and even explore dynamic motions beyond walking [13,14,15].

While many studies have focused on the locomotion control of legged robots, there has been relatively little research on fall recovery. Although many studies have shown the impressive locomotion performance of legged robots, falls are inevitable for legged robots. In the case of many legged robots, it can be seen that they recover from a fall while moving along a pre-defined trajectory such as HyQ2Max [16] or Unitree Go1 [17]. However, designing a trajectory for fall recovery requires significant engineering efforts and is limited because it is challenging to consider various situations.

Fall recovery for quadruped robots can be broadly divided into two stages: self-righting and standing up. Lee et al. [18] successfully performed fall recovery using a hierarchical behavior-based controller. They trained individual neural networks for each stage, and after training each network, they trained a behavior selector network that switches the networks according to the situation.

On the other hand, some studies have explored the fall recovery of robots using a single neural network. Smith et al. [19] aimed to train a neural network that enables robot walking using a real robot, not a simulation. They used a reset policy to enable the robot to get up on its own when it fell during training. The reset policy was trained through simulation. First, they rewarded the agent for the right side to be up, and if the robot was upright within the threshold, they added an imitation reward to encourage the robot to stand. Nahrendra et al. [20] applied the structure from [21] and presented a study to obtain implicit information containing the surrounding terrain of a quadrupedal robot using the history of observation, and used this to recover from the robot’s fall in various environments.

Previous studies have achieved the successful fall recovery of robots, but either required the use of multiple networks or focused on recovering the robot’s orientation rather than standing up.

In this study, we propose a method using a single neural network to enable a robot to regain its orientation and stand up. The contributions of this work are summarized as follows:

- We propose a framework that condenses the knowledge about fall recovery, which was previously achieved using multiple networks in a hierarchical structure, into a single neural network.
- Using the single network, we demonstrate that a real quadruped robot can successfully recover from random postures (including completely inverted postures) or disturbance-induced falls.

## 2. Materials and Methods

**Figure 1:**
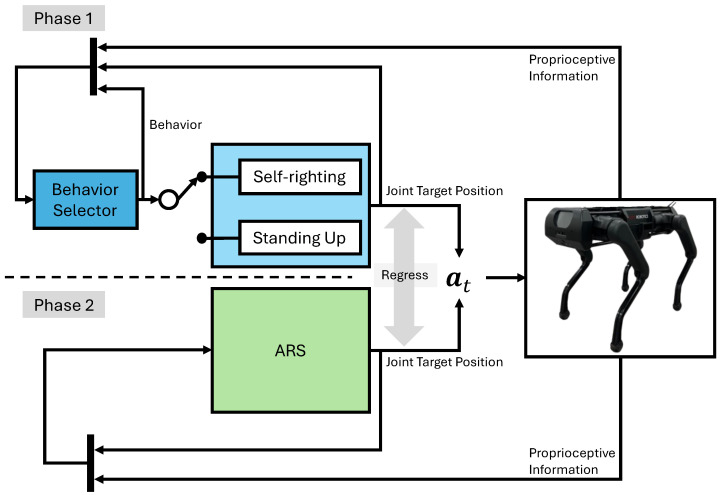
The proposed method, ARS, consists of two phases. In phase 1, fall recovery is executed by learning a self-righting and standing-up policy and training a behavior selector that chooses between them based on the state. In phase 2, a single neural network is trained to recover from a fall using reinforcement learning, incorporating imitation loss to replicate the actions of the hierarchical behavior-based controller learned in phase 1.

This work aims to develop a fall recovery controller using a single neural network. To train the neural network that enables the quadruped robot’s falling recovery, we used Deep RL, specifically model-free Deep RL. An overview of our framework is illustrated in Figure 1.

### 2.1. Reinforcement Learning

To address the robot fall recovery problem using RL, we modeled the problem as a Markov decision process (MDP). An MDP is defined by a state space *S*, an action space *A*, a reward function $R(s_{t+1}|s_{t},a_{t})$, a transition probability $P(s_{t+1}|s_{t},a_{t})$, and a discount factor γ∈[0,1). A learning agent uses its policy $\pi (a_{t}|s_{t})$ to determine the action $a_{t}$ based on the current state $s_{t}$, and receives a reward $r_{t}$ from the environment. The agent’s goal is to find the optimal policy $\pi^{*}$ that maximizes the discounted sum of rewards, expressed as follows:

(1) $$J\left(\pi \right)=E_{\pi }\left[\sum_{t=0}^{\infty }\gamma^{t}r_{t}\right]$$

To find the optimal policy, we chose to use Proximal Policy Optimization (PPO) [22] among the policy gradient methods because it has been shown in various studies [21,23,24,25] to solve high-dimensional continuous MDPs successfully. The loss function of PPO is as follows:

(2) $$L_{ppo}\left(\theta \right)=E_{\pi }\left[\min(r\left(\theta \right)A_{t},clip\left(r\left(\theta \right),1-\epsilon ,1+\epsilon \right)A_{t})\right]$$

where $r\left(\theta \right)=\pi_{\theta }\left(a|s\right)/\pi_{\theta_{old}}\left(a|s\right)$ is an importance sampling term, $A_{t}$ is an advantage function, and ϵ is a clipping ratio that is a hyperparameter of PPO. For a more detailed description of PPO, refer to [22].

### 2.2. Hierarchical Behavior-Based Controller

To achieve robot fall recovery with a single network, we divided the process into two phases instead of learning everything with a single network. In the first phase, we trained the hierarchical behavior-based controller ($\pi_{base}$). We exploited the method of [18] to train a network for each task and a behavior selector that switches the network according to the current state of the robot. Subsequently, we confirmed that $\pi_{base}$ performs effectively on the actual robot.

### 2.3. Imitation Loss

In the second phase, a single network ($\pi_{single}$) was trained to imitate the action of $\pi_{base}$. As mentioned in [23], state–action pairs for imitation learning can be collected using $\pi_{base}$, but in this case, only good data will be collected in the dataset. If imitation learning is performed using this dataset, the trained $\pi_{single}$ is not robust against situations that deviate from those experienced by $\pi_{base}$. Therefore, a simulation was performed using $\pi_{single}$, and a loss was applied to imitate the action of $\pi_{base}$. Hence, we trained the student network from scratch by adding the imitation loss ($L_{imitation}$) to the PPO loss ($L_{PPO}$) as follows:

(3) $$L_{revised}=L_{PPO}+\beta \;L_{imitation}$$

(4) $$L_{imitation}={∥\pi_{base}\left(\cdot |s\right)-\pi_{single}\left(\cdot |s\right)∥}_{2}^{2}$$

where β is a weight to balance the expected return of the policy and imitating the action of the hierarchical behavior-based controller, $\pi_{base}\left(\cdot |s\right)$ is the action from the hierarchical behavior-based controller, and $\pi_{single}\left(\cdot |s\right)$ is the action from the single network, corresponding to ARS in Figure 1.

### 2.4. Observation and Action Space

The observation space, $o_{t}$, is an 45×1 vector consisting of the following:

$$o_{t}=\left[v_{t}\;\omega_{t}\;g_{t}\;q_{t}\;{\dot{q}}_{t}\;a_{t-1}\right]$$

where $v_{t}$ and $\omega_{t}$ are the base linear and angular velocity, $g_{t}$ is a projected gravity vector onto the robot’s body frame, and all the vectors described above are 3 × 1 vectors. $q_{t}$ and ${\dot{q}}_{t}$ are the joint positions and velocities, and $a_{t-1}$ is the previous actions, and all are 12 × 1 vectors.

In many studies of legged robots using RL, standing joint positions are used as nominal joint positions when mapping network outputs to joint target values [7,21,25] to accelerate the learning process in the initial phase. However, in our case, like in [18], to promote exploration in self-righting situations, we did not use the nominal joint position but used the current joint position of the robot, $q_{t}$. The network output, $a_{t}$, is a 12×1 vector, and it is clipped to the range [−5, +5] and multiplied by the scaling factor k=0.1. The joint target value, $q_{t}^{des}$, is expressed as follows:

(5) $$q_{t}^{des}=q_{t}+k\;a_{t}.$$

The action clip range and scaling factor were determined heuristically. The joint target values ($q_{t}^{des}$) are sent to the motor drivers of each joint and tracked using proportional-derivative (PD) control.

### 2.5. Reward Functions

The reward function is defined as shown in Table 1. The reward function is defined to ensure that the robot can stably recover from a fall and that the learned network can be applied to actual robots. Among the reward functions, the joint position, orientation, and base height reward were applied to ensure the stability of the robot, and torque rewards were applied to enable energy-efficient movements. In addition, rewards for the joint velocity, acceleration, action magnitude, and hip-joint torque limit were applied to ensure smoothness and sim-to-real transfer. In Table 1, $q_{task}$ is the joint target value for each task. The sitting posture was used in the self-righting task, and the standing posture was used in the standing task and ARS training.

The hip torque limit reward is related to the hardware characteristics, AiDIN-VIII, developed by Aidin Robotics [26]. In real robots, the hip and knee links are connected in parallel, but this cannot be implemented in a simulation, so a model connected in series is used. Therefore, the driving torque of the simulation and actual robots requires the following transformation.

(6) $$\tau_{real}=T\tau_{sim}$$

where $\tau ={\left[\tau_{scap}\;\tau_{hip}\;\tau_{knee}\right]}^{T}$ is a 3×1 joint torque vector for each leg and T is given by the following:

(7) $$T=\left[\begin{array}{ccc} 1 & 0 & 0\\ 0 & 1 & -1\\ 0 & 0 & 1 \end{array}\right]$$

For this reason, in addition to ensuring that each joint torque does not exceed the torque limit, restrictions are needed to ensure that the absolute value of the difference between the hip joint torque and knee joint torque does not exceed the torque limit. We found that the sim-to-real performance differed significantly with and without this term, and we believe that this contributed significantly to making sim-to-real possible. The impact of the hip torque limit reward on sim-to-real is discussed in the ablation study in Section 3.6.

The rewards used are different for each network being trained. The reward at time *t* is defined as the weighted sum of each reward function as $r_{t}=w_{q}r_{q}+w_{ori}r_{ori}+w_{h}r_{h}+w_{\tau }r_{\tau }+w_{a}r_{a}+w_{v}r_{v}+w_{acc}r_{acc}+w_{lim}r_{lim}$.

When training the ARS and standing-up policy, the weights were 1.0, −5.0, 1.0, −0.00001, −0.025, −1.0, −0.000001, and −1.0. When training the self-righting policy, $w_{h}$ was 0 and $w_{q}$ was 8, and when training the behavior selector, $w_{q}$ was 0.

A kernel function similar to [7] was used for the joint position. The kernel function *K*: R→[0,0.25) is defined as $K\left(e,\alpha \right)=1/\left(e^{\alpha e}+2+e^{-\alpha e}\right)$, where *e* is the error amount, and α is a positive number that controls how sensitive it is to error.

### 2.6. Sim-to-Real Additions

To facilitate a successful sim-to-real transfer, we employed domain randomization, a widely used method [27,28,29], to ensure that agents are exposed to a variety of situations during training, thereby enhancing their robustness. Additionally, we introduced a certain noise level to the observations to make the agent resilient to sensor noise when deployed on an actual robot. We further increased the agent’s robustness by applying random forces at regular intervals during training. The range of domain randomization and noise parameters used in training is detailed in Table 2. In order to apply random pushes to the robot during learning in the simulation, the method of Rudin et al. [24] was used. During the learning process, the robot’s base was accelerated up to ±2 m/s in both the x and y directions every 10 s.

## 3. Results

### 3.1. Simulation

For the simulation, the Legged Gym and RSL-RL [24] environment configured in Isaac Gym [30] was used. In our case, we trained 4096 agents, each running in parallel in 24 steps to collect data and divide the data into four mini-batches. During training, the standing-up and self-righting policies each performed 5000 iterations, and the behavior selector and ARS performed 3000 iterations.

To train the neural network, PPO was used. All networks were trained at 100 Hz, and simulations were run at 400 Hz. The training was conducted on a desktop PC equipped with an AMD Ryzen7 3700X CPU, 32 GB RAM, and an Nvidia RTX 2070 super GPU.

To sample the initial states of the robot, the robot was dropped from the air in a random state. Postures in which the robot’s orientation is relatively upright were sampled to train the standing-up policy. To increase the efficiency of the initial phase of the learning process, when training the standing-up policy and ARS, the initial state was sampled from a multivariate Gaussian distribution with the standing configuration as the mean with a probability of 10%. The only termination condition in all training processes was time.

### 3.2. Network Structure

The final performance of the RL is affected by the size of the neural network. Xie et al. [31] showed that learning becomes faster as the network size increases, and Nahendra et al. [21] and Zhang et al. [32] successfully controlled a quadruped robot using hidden layers with 512, 256, and 128 neurons. By referring to previous studies, all networks were parameterized as a multi-layer perceptron (MLP) with three hidden layers, each with 512, 256, and 128 neurons. Exponential Linear Unit (ELU) was used as an activation function when passing between hidden layers. For the behavior selector, Softmax was applied to the output. Table 3 lists the PPO hyperparameters and coefficient β of $L_{imitation}$.

### 3.3. Hardware and Deployment

In this study, we experimented using AiDIN-VIII. The robot was equipped with an inertial measurement unit (IMU), a tracking camera, and joint encoders, which provide information about the robot’s orientation, body velocity, joint positions, and joint velocities. Each leg had three degrees of freedom and consisted of the scap(hip abduction/adduction), hip (hip flexion/extension), and knee (knee flexion/extension) joints. Each actuator had a peak torque of 89.8 Nm and a maximum speed of 10 rad/s. The robot weighed approximately 60 kg.

The trained policy was run on the on-board PC built into the robot. The trained network was evaluated at 100 Hz, and communication between the onboard PC and motor driver was performed at 1 kHz. To track the desired joint angles, we used a PD controller with the proportional and derivative gains set to $K_{p}$ = 200 and $K_{d}$ = 3, respectively, which was identical to the simulation.

### 3.4. Results

The performance of the trained network was verified on a real robot and in simulation. First, the robot was dropped from a certain height to a random posture in a simulation environment, and the fall recovery performance and computation time were compared. In order to compare how smoothly the fall recovery was performed, the standard deviation of the action rate and joint acceleration were compared. Here, the action rate represents the difference in the actions at time step *t* and time step t−1. We compared a total of four policies in our simulations, each of which is as follows:

- Baseline ($\pi_{base}$): A controller with a hierarchy of a behavior selector, self-righting policy, and standing-up policy, trained using the pipeline of Lee et al. [18].
- ImitationNet ($\pi_{imit}$): A policy trained using only imitation loss to mimic the behavior of $\pi_{base}$.
- ARS ($\pi_{ars}$): A policy trained with the sum of the imitation loss and PPO loss as described in Equation (3).
- SingleNet ($\pi_{LS}$): A policy trained along the pipeline of Smith et al. [19], which only rewards the policy for the robot’s orientation, and then provides an additional reward when the robot becomes upright to encourage it to follow the robot’s standing pose.

To compute the imitation loss, we used Data Aggregation (DAgger) [33]. $\pi_{imit}$ and $\pi_{ars}$ obtained the action of $\pi_{base}$ for the states visited during the learning process and imitated it.

It was considered successful when the body height exceeds 0.55 m within 5 s of the starting fall recovery. The baseline showed the best fall recovery success rate. ImitationNet and ARS, which were trained to imitate the baseline using three neural networks with a single neural network, also showed similar fall recovery success rates, but SingleNet performed worse than the other three policies. SingleNet only rewards orientation when the robot is not upright, so it tries to make it upright as fast as possible. This causes very aggressive movements, which can be confirmed through the standard deviation of the action rate and joint acceleration. Such aggressive movements can cause damage when applied to actual hardware. Since ImitationNet was trained to imitate the baseline, the standard deviations of the action rate and joint acceleration were similar to those of the baseline. On the other hand, ARS showed a decrease in the standard deviations of the action rate and joint acceleration compared to the baseline, indicating that it generates a smoother trajectory for fall recovery. The baseline first decides which action to take for fall recovery through an action selector, and then acts upon the chosen action through a policy. Therefore, neural network inference is required twice. However, the other three networks perform crash recovery in just one inference, which significantly reduces the computation time. The results are shown in Table 4.

**Figure 2:**
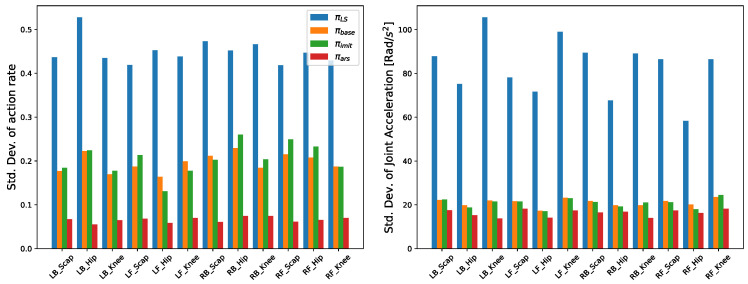
Comparison of standard deviation of action rate and joint acceleration during fall recovery in a simulation.

**Figure 3:**
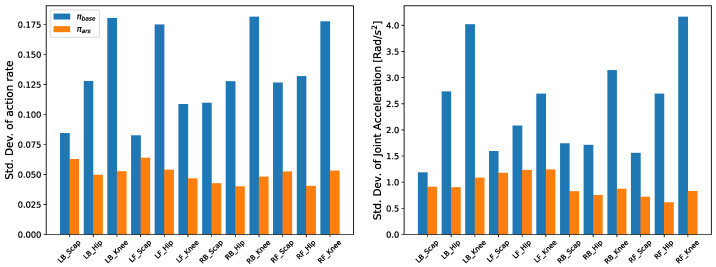
Comparison of standard deviation of action rate and joint acceleration during fall recovery in real robot experiment.

The standard deviation of the action rate and joint acceleration of each joint for each policy when the robot started from the same posture, recovered from a fall, and encountered the same disturbance at regular intervals are shown in Figure 2. In the same fall recovery scenario, the proposed method demonstrates improvements over the comparison policies as illustrated in Figure 2. When we performed an experiment to recover from a fall by kicking the robot, we compared the standard deviations of the action rate and joint accelerations of the Baseline and ARS. As can be seen in Figure 3, ARS shows smaller standard deviations of action rates and joint accelerations across all joints. We believe that the improvement stems from the fact that the hierarchical behavior-based controller inevitably results in a higher action rate when switching between policies. This is because the self-righting policy and the standing-up policy are trained to achieve different objectives, leading to divergent outputs in the same situation. This phenomenon was observed during our experiments with the robot, as demonstrated in Video S1 in the Supplementary Materials.

**Figure 4:**
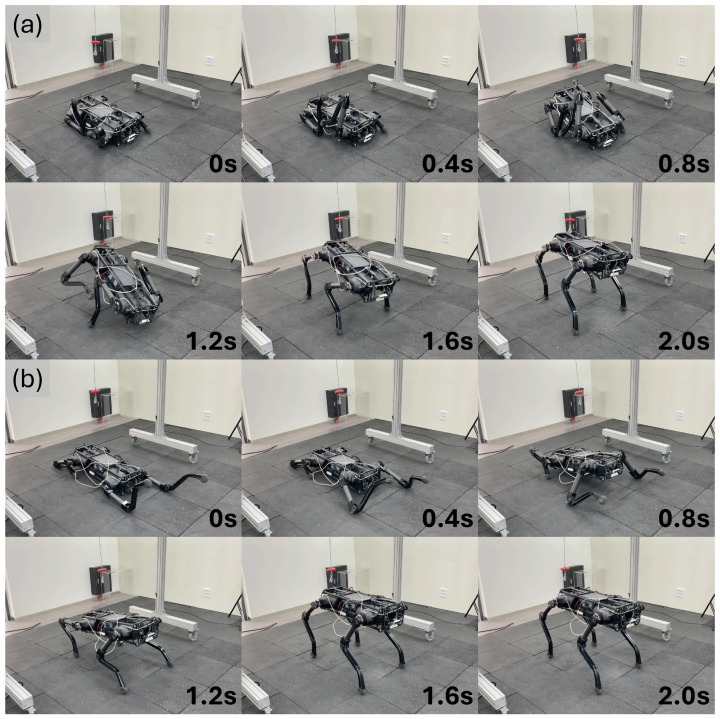
Fall recovery from different postures. (**a**,**b**) A snapshot of the experiment showcasing the robot initialized in random poses and recovering from a fall.

**Figure 5:**
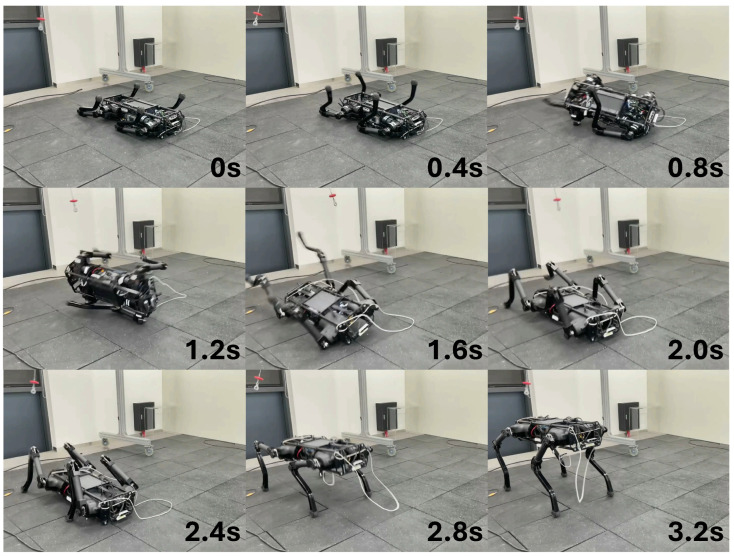
Fall recovery from a completely inverted posture.

**Figure 6:**
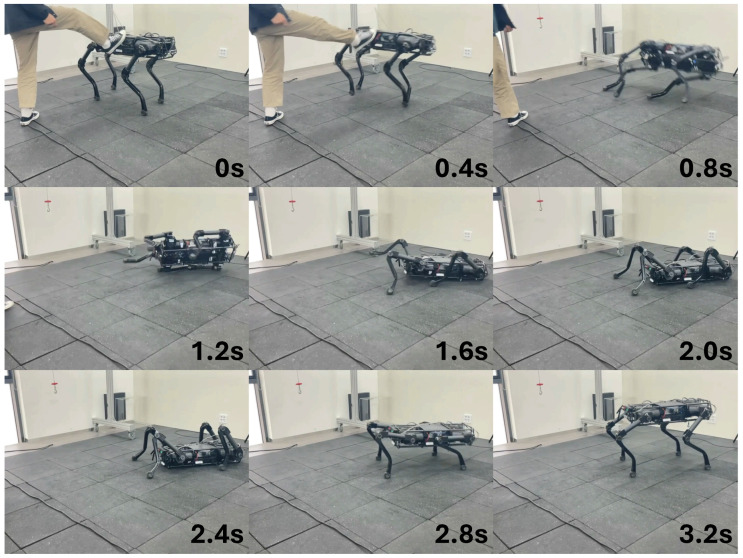
Snapshot of AiDIN-VIII recovering after being kicked using a single neural network trained with the proposed framework.

The performance of ARS on the real robot was verified. First, we tested whether the robot could recover from a fall in various random positions. The fall recovery experiment conducted after the robot was dropped into a random posture is shown in Figure 4. In situations where the forelegs and hind legs overlapped, the robot would folded the scap joints of the forelegs to allow the hind legs to pass and then stand up (Figure 4a), or, if both forelegs were extended forward, it would pull the forelegs forward to create space between the ground and the body to allow the hind legs to move and then stand up (Figure 4b). We tested whether fall recovery was possible even when the robot was completely turned over, and we could see that fall recovery was successful, as shown in Figure 5. In this situation, the robot bended the scap joints of all legs to create space between its body and the ground, and bended the hip joints of its two right legs to push the ground and create a force to rotate its body. When its body rotated and became upright, it stood up after touching the ground with all four feet, thereby recovering from the fall state. Second, we tested whether the robot can recover stably even when it falls due to a disturbance while standing. As shown in Figure 6, when the robot fell over due to external disturbance, it lowered the height of its body by rotating the hip joints of the two legs in contact with the ground, and after the body collided with the ground, it lifted the opposite legs so that the body can land on the ground without flipping over. When the body became stable, it placed all four feet on the ground and recovered its posture.

### 3.5. Unseen Environment

**Figure 7:**
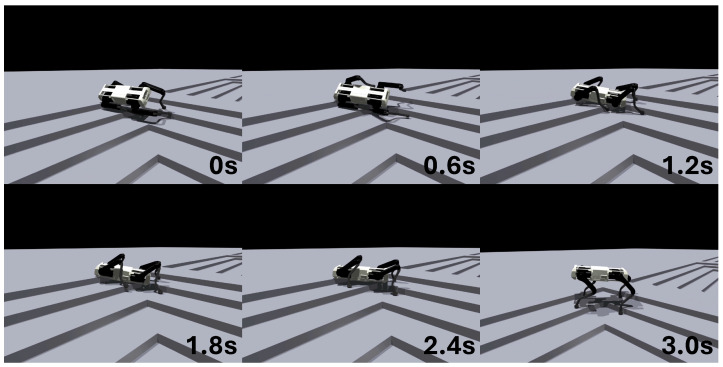
Experiment in a 0.1 m high stair environment that was not encountered during the training of ARS.

The fall recovery controller, despite being trained exclusively in flat-terrain environments, demonstrated the ability to recover from falls even on uneven terrains with height variations, such as stairs. When the performance of the trained controller was evaluated on stair terrains with height differences of 0.025, 0.05, 0.075, and 0.1 m, it achieved success rates of 85%, 80%, 74%, and 58%, respectively. A snapshot of the fall recovery controller’s movement on a stair terrain with a height difference of 0.1 m in the simulation is shown in Figure 7.

### 3.6. Ablation Study

We conducted ablation studies for two cases. The first compares the learning performance of a single neural network with and without imitation loss, and the second compares sim-to-real performance with and without a hip torque limit reward.

**Figure 8:**
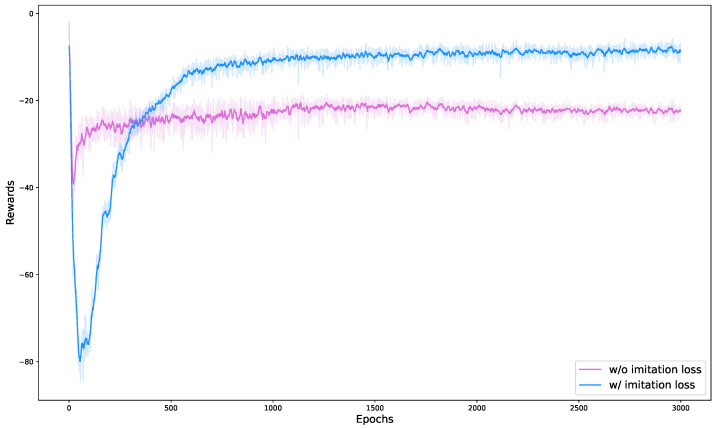
This graph depicts the learning performance with and without imitation loss. The blue line is when trained using imitation loss, and the pink line is when trained with a single neural network without imitation loss. The line represents the moving average of the reward, while the lighter color in the background represents the raw data of the reward.

We compared the learning results with and without limitation loss as can be seen in Figure 8. In our reward setting, fall recovery using a single neural network was not trained without limitation loss. Although Smith et al. [19] achieved fall recovery using a single neural network, it was not practical for application to medium-sized robots like ours, which are approximately five times heavier and larger than theirs. We attempted to modify their approach by incorporating the joint velocity limit reward we used, but were unable to successfully train the network for fall recovery. Through the learning performance graph with and without imitation loss, it can be inferred that there is a problem of falling into a local minimum when training a robot’s fall recovery using a single neural network and that this can be overcome by using imitation loss.

**Figure 9:**
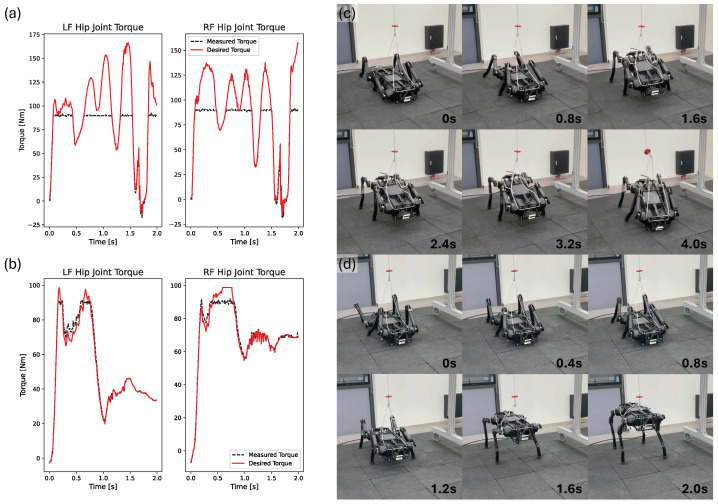
The red solid line represents the desired torque calculated through the PD controller, while the black dashed line represents the actual torque calculated using the current measured from the robot’s motor driver. Graph (**a**) displays the results of applying the policy learned without considering a hip torque limit reward, whereas graph (**b**) illustrates the results of applying the policy learned while considering a hip torque limit reward. Snapshots (**c**,**d**) depict experimental snapshots using a real robot without and with the hip torque limit reward, respectively.

Next, we compared sim-to-real transfer performance with and without a hip torque limit reward. In the simulation, both cases with and without a reward for limiting hip torque achieved successful fall recovery. However, there were clear differences in the performance of the actual robot. In Figure 9, the reader can see the difference in sim-to-real performance depending on there being a hip torque limit reward or not. It can be seen that the policy trained without a hip torque limit reward generated movements that require excessive torque on the hip joint of the robot, and as a result, the robot fell, as shown in Figure 9a,c. On the other hand, it can be seen in Figure 9b,d that a trained policy, including a hip torque limit reward, produced trajectories that do not require excessive torque on the hip joint and led to successful fall recovery. When there was no hip torque limit reward, the robot tried to raise its body with its center of gravity quickly tilted forward, imposing excessive torque on the hip joints of the front legs. However, when there was a hip torque limit reward, it happened after the center of gravity was moved more to the center. As a result, the force was not excessively applied to the front legs and was evenly distributed to all four legs.

## 4. Discussion

In this paper, we propose ARS, a single-neural network framework trained using reinforcement learning, which enables fall recovery in quadrupedal robots. The trained network can perform fall recovery, which can be divided into two tasks (self-righting and standing up), and tests were performed using simulations and a real robot to verify the network’s performance. The results show that the robot successfully recovered its posture from various postures, including a completely inverted posture, and from falling due to a disturbance.

The unified controller successfully recovered from a fall in most scenarios, even when subjected to strong forces that caused the robot to fall or flip over. However, it was not able to recover in all cases. One notable failure scenario occurred when interference between leg links took place. We believe that this limitation arose because collision information between the robot’s links could not be utilized effectively in the simulation environment we used. As a result, this factor was not reflected in the reward function. We believe that incorporating a reward for self-collision avoidance, as suggested by Lee et al. [18], could enable the training of a more robust and effective recovery controller.

Self-righting is a very important behavior for recovering from a fall, and this phenomenon has been observed in various animals [34,35]. Therefore, we believe that the method we propose is not limited to quadrupedal robots, and can be extended to legged robots that can recover from a fall through self-righting and standing-up.

The present method does not use any information about the robot’s surrounding environment (e.g., a point cloud), but only uses the robot’s proprioceptive information. In future work, we believe that research on fall recovery on terrain other than flat ground can advance by utilizing information about the surrounding environment or applying a curriculum that raises the difficulties of the environment. Additionally, efficient sampling during the learning process will be possible by efficiently sampling the initial state of the robot using methods such as that in [36].

## Figures and Tables

**Table 1:** Reward functions.

Reward	Expression
Joint Position ($r_{q}$)	$K(|q_{task}-q_{t}|,\alpha )$
Orientation ($r_{ori}$)	${∥\left[0,0,-1\right]}^{T}-g_{t}{∥}_{2}^{2}$
Base Height ($r_{h}$)	1 if $h_{t}<h^{des}$, otherwise 0
Joint Torque ($r_{\tau }$)	${∥\tau ∥}_{2}^{2}$
Action Magnitude ($r_{a}$)	$∥a_{t}{∥}_{2}^{2}$
Joint Velocity ($r_{v}$)	$\sum_{i=1}^{12}c_{i}$, where
	if $|{\dot{q}}_{i}|>{\dot{q}}_{limit},\;c_{i}=\left(\right|{\dot{q}}_{i}{\left|-{\dot{q}}_{limit}\right)}^{2}$, otherwise 0
Joint Acceleration ($r_{acc}$)	$∥\ddot{q}{∥}_{2}^{2}$
Hip Torque Limit ($r_{lim}$)	$\sum_{i=1}^{leg}c_{i}$, where
	$c_{i}=1$ if $|\tau_{i,hip}-\tau_{i,knee}|>\tau_{limit}$, otherwise 0

**Table 2:** Domain randomization and noise range.

Parameter	Range
Base Mass	[−5, 5] kg
Joint Position	[−0.01, 0.01] rad
Joint Velocity	[−1.5, 1.5] rad/s
Base Linear Velocity	[−0.1, 0.1] m/s
Base Angular Velocity	[−0.2, 0.2] rad/s
Friction	[0.5, 1.25]

**Table 3:** Hyperparameters.

Parameter	Value
Horizon	24
Discount factor	0.995
GAE discount factor	0.95
Clipping range	0.2
Number of epochs	5
Learning rate	Adaptive [24]
	0.0003 (behavior selector)
Mini-batch size	24576
Entropy coeff.	0.01
α for kernel function	2
Imitation coeff. β	0.05

**Table 4:** Fall recovery performance compare in a simulation environment.

Policy	Success Rate (%)	Computation Time (μs)	Standard Deviation	
			Action Rate	Joint Acceleration
Baseline	**90.4 ± 1.90**	519 ± 18.98	0.197	21.15
ImitationNet	90.0 ± 1.04	250 ± 3.70	0.206	20.91
ARS	90.0 ± 0.77	**247 ± 3.54**	**0.066**	**16.36**
SingleNet	83.1 ± 0.68	247 ± 3.64	0.451	83.91

## Data Availability

Data are contained within the article.

## Supplementary Materials

The following supporting information can be downloaded at: https://www.mdpi.com/article/10.3390/biomimetics9120749/s1, Video S1: ARS.

## References

[1] Hutter M., Hoepflinger M.A., Gehring C., Bloesch M., Remy C.D., Siegwart R. (2013). Hybrid Operational Space Control for Compliant Legged Systems. Robotics: Science and Systems VIII.

[2] Di Carlo J., Wensing P.M., Katz B., Bledt G., Kim S. Dynamic locomotion in the mit cheetah 3 through convex model-predictive control. Proceedings of the 2018 IEEE/RSJ International Conference on Intelligent Robots and Systems (IROS).

[3] Kim D., Di Carlo J., Katz B., Bledt G., Kim S. (2019). Highly dynamic quadruped locomotion via whole-body impulse control and model predictive control. arXiv.

[4] Chignoli M., Kim D., Stanger-Jones E., Kim S. The MIT humanoid robot: Design, motion planning, and control for acrobatic behaviors. Proceedings of the 2020 IEEE—RAS 20th International Conference on Humanoid Robots (Humanoids).

[5] Tan J., Zhang T., Coumans E., Iscen A., Bai Y., Hafner D., Bohez S., Vanhoucke V. (2018). Sim-to-real: Learning agile locomotion for quadruped robots. arXiv.

[6] https://www.ghostrobotics.io/.

[7] Hwangbo J., Lee J., Dosovitskiy A., Bellicoso D., Tsounis V., Koltun V., Hutter M. (2019). Learning agile and dynamic motor skills for legged robots. Sci. Robot..

[8] Hutter M., Gehring C., Jud D., Lauber A., Bellicoso C.D., Tsounis V., Hwangbo J., Bodie K., Fankhauser P., Bloesch M. Anymal—A highly mobile and dynamic quadrupedal robot. Proceedings of the 2016 IEEE/RSJ International Conference on Intelligent Robots and Systems (IROS).

[9] Miki T., Lee J., Hwangbo J., Wellhausen L., Koltun V., Hutter M. (2022). Learning robust perceptive locomotion for quadrupedal robots in the wild. Sci. Robot..

[10] Duan H., Pandit B., Gadde M.S., van Marum B.J., Dao J., Kim C., Fern A. (2023). Learning vision-based bipedal locomotion for challenging terrain. arXiv.

[11] Grandia R., Jenelten F., Yang S., Farshidian F., Hutter M. (2023). Perceptive locomotion through nonlinear model-predictive control. IEEE Trans. Robot..

[12] Jenelten F., He J., Farshidian F., Hutter M. (2023). DTC: Deep Tracking Control—A Unifying Approach to Model-Based Planning and Reinforcement-Learning for Versatile and Robust Locomotion. arXiv.

[13] Li C., Vlastelica M., Blaes S., Frey J., Grimminger F., Martius G. Learning agile skills via adversarial imitation of rough partial demonstrations. Proceedings of the Conference on Robot Learning.

[14] Zhuang Z., Fu Z., Wang J., Atkeson C.G., Schwertfeger S., Finn C., Zhao H. Robot Parkour Learning. Proceedings of the Conference on Robot Learning.

[15] Hoeller D., Rudin N., Sako D., Hutter M. (2024). Anymal parkour: Learning agile navigation for quadrupedal robots. Sci. Robot..

[16] Semini C., Barasuol V., Goldsmith J., Frigerio M., Focchi M., Gao Y., Caldwell D.G. (2016). Design of the hydraulically actuated, torque-controlled quadruped robot HyQ2Max. IEEE ASME Trans. Mechatron..

[17] https://www.unitree.com.

[18] Lee J., Hwangbo J., Hutter M. (2019). Robust recovery controller for a quadrupedal robot using deep reinforcement learning. arXiv.

[19] Smith L., Kew J.C., Peng X.B., Ha S., Tan J., Levine S. Legged robots that keep on learning: Fine-tuning locomotion policies in the real world. Proceedings of the 2022 International Conference on Robotics and Automation (ICRA).

[20] Nahrendra I., Oh M., Yu B., Lim H., Myung H. (2023). Robust recovery motion control for quadrupedal robots via learned terrain imagination. arXiv.

[21] Nahrendra I.M.A., Yu B., Myung H. DreamWaQ: Learning robust quadrupedal locomotion with implicit terrain imagination via deep reinforcement learning. Proceedings of the 2023 IEEE International Conference on Robotics and Automation (ICRA).

[22] Schulman J., Wolski F., Dhariwal P., Radford A., Klimov O. (2017). Proximal policy optimization algorithms. arXiv.

[23] Kumar A., Fu Z., Pathak D., Malik J. (2021). RMA: Rapid Motor Adaptation for Legged Robots. Robotics: Science and Systems XVII.

[24] Rudin N., Hoeller D., Reist P., Hutter M. Learning to walk in minutes using massively parallel deep reinforcement learning. Proceedings of the Conference on Robot Learning.

[25] Ji G., Mun J., Kim H., Hwangbo J. (2022). Concurrent training of a control policy and a state estimator for dynamic and robust legged locomotion. IEEE Robot. Autom. Lett..

[26] https://www.aidinrobotics.co.kr.

[27] Siekmann J., Green K., Warila J., Fern A., Hurst J. (2021). Blind bipedal stair traversal via sim-to-real reinforcement learning. arXiv.

[28] Xie Z., Da X., Babich B., Garg A., de Panne M.v. (2022). Glide: Generalizable quadrupedal locomotion in diverse environments with a centroidal model. Proceedings of the International Workshop on the Algorithmic Foundations of Robotics.

[29] Haarnoja T., Moran B., Lever G., Huang S.H., Tirumala D., Humplik J., Wulfmeier M., Tunyasuvunakool S., Siegel N.Y., Hafner R. (2024). Learning agile soccer skills for a bipedal robot with deep reinforcement learning. Sci. Robot..

[30] Makoviychuk V., Wawrzyniak L., Guo Y., Lu M., Storey K., Macklin M., Hoeller D., Rudin N., Allshire A., Handa A. (2021). Isaac Gym: High Performance GPU-Based Physics Simulation For Robot Learning. arXiv.

[31] Xie Z., Clary P., Dao J., Morais P., Hurst J., Panne M. Learning locomotion skills for cassie: Iterative design and sim-to-real. Proceedings of the Conference on Robot Learning.

[32] Zhang C., Rudin N., Hoeller D., Hutter M. (2023). Learning agile locomotion on risky terrains. arXiv.

[33] Ross S., Gordon G., Bagnell D. A reduction of imitation learning and structured prediction to no-regret online learning. Proceedings of the Fourteenth International Conference on Artificial Intelligence and Statistics.

[34] Li C., Kessens C.C., Young A., Fearing R.S., Full R.J. (2016). Cockroach-inspired winged robot reveals principles of ground-based dynamic self-righting. Proceedings of the 2016 IEEE/RSJ International Conference on Intelligent Robots and Systems (IROS).

[35] Ana G., Ljiljana T., Ana I. (2015). Geometry of self righting: The case of Hermann’s tortoises. Zool. Anz. A J. Comp. Zool..

[36] Messikommer N., Song Y., Scaramuzza D. (2023). Contrastive Initial State Buffer for Reinforcement Learning. arXiv.

